# Supplementary material for: The relationships between box turtle gut microbiomes and personality
Source: PLoS One. 2025 Dec 19;20(12):e0339132. doi: 10.1371/journal.pone.0339132 (PMC12716703; doi:10.1371/journal.pone.0339132)
Supplement: S1 Fig — Males (n = 6) and n.a. (n = 2) (Kruskal-Wallis pairwise test, p = 0.045500). (DOCX) [file pone.0339132.s001.docx]

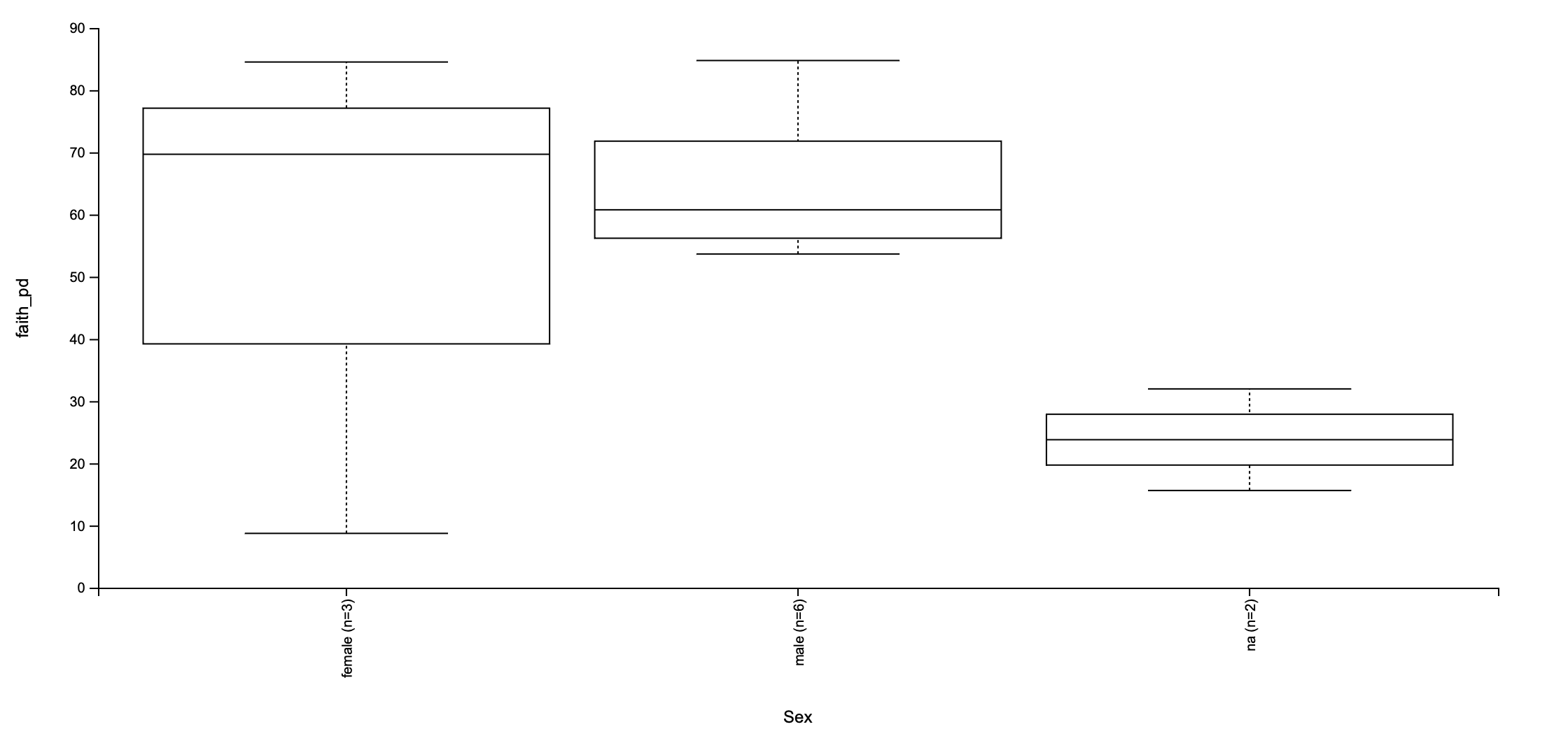


**S1 Fig.** **Alpha diversity (Faith’s phylogenetic diversity) of bacterial communities between sexes.** Males (n=6) and n.a. (n=2) (Kruskal-Wallis pairwise test, p = 0.045500).
