# Supplementary material for: The relationships between box turtle gut microbiomes and personality
Source: PLoS One. 2025 Dec 19;20(12):e0339132. doi: 10.1371/journal.pone.0339132 (PMC12716703; doi:10.1371/journal.pone.0339132)
Supplement: S2 Fig — (DOCX) [file pone.0339132.s002.docx]

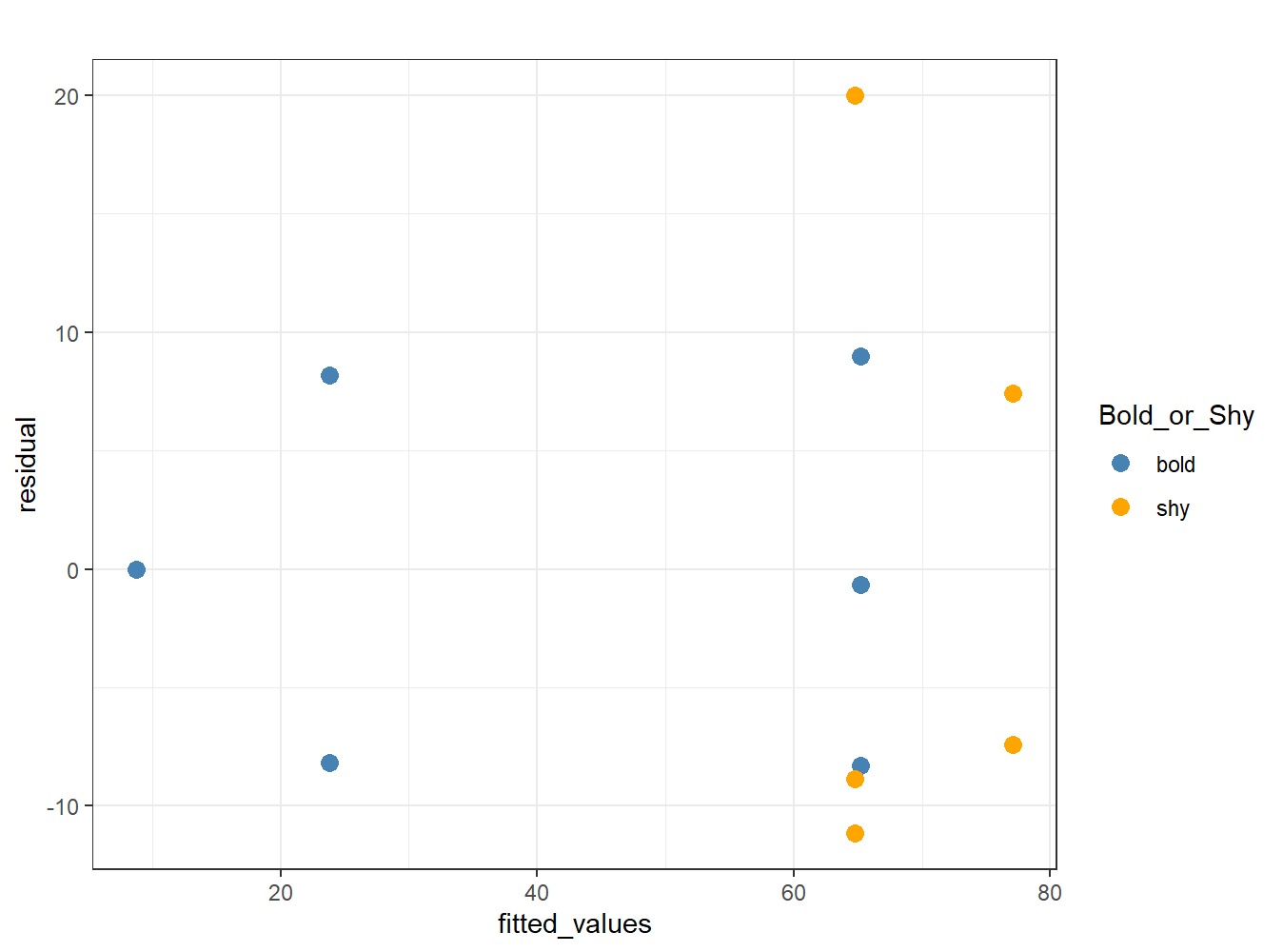

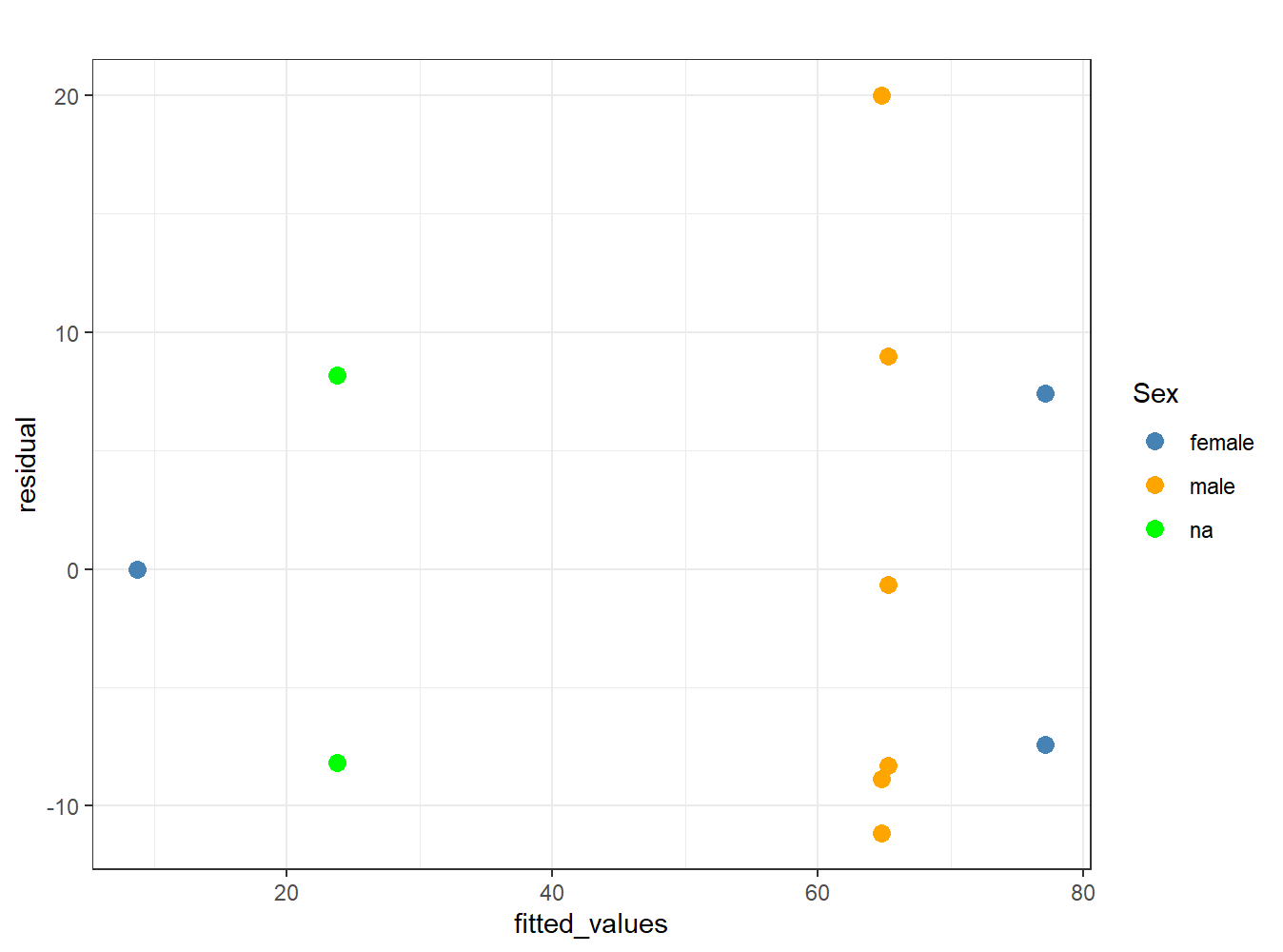


**S2 Fig. ANOVA (Faith’s phylogenetic diversity) of bacterial communities between bold and shy turtles to sex**. sum_sq = 2186.8, df = 1, F = 113.191, PR(>F) = 0.0109.
