# Supplementary material for: The relationships between box turtle gut microbiomes and personality
Source: PLoS One. 2025 Dec 19;20(12):e0339132. doi: 10.1371/journal.pone.0339132 (PMC12716703; doi:10.1371/journal.pone.0339132)
Supplement: S3 Fig — (A) skin samples (R2 = 0.11447, p = 0.595), (B) Oral samples (R2 = 0.094497, p = 0.734), and (C) cloacal samples (R2 = 0.1137, p = 0.894). Shaded ellipses represent the 95% confidence interval. (DOCX) [file pone.0339132.s003.docx]

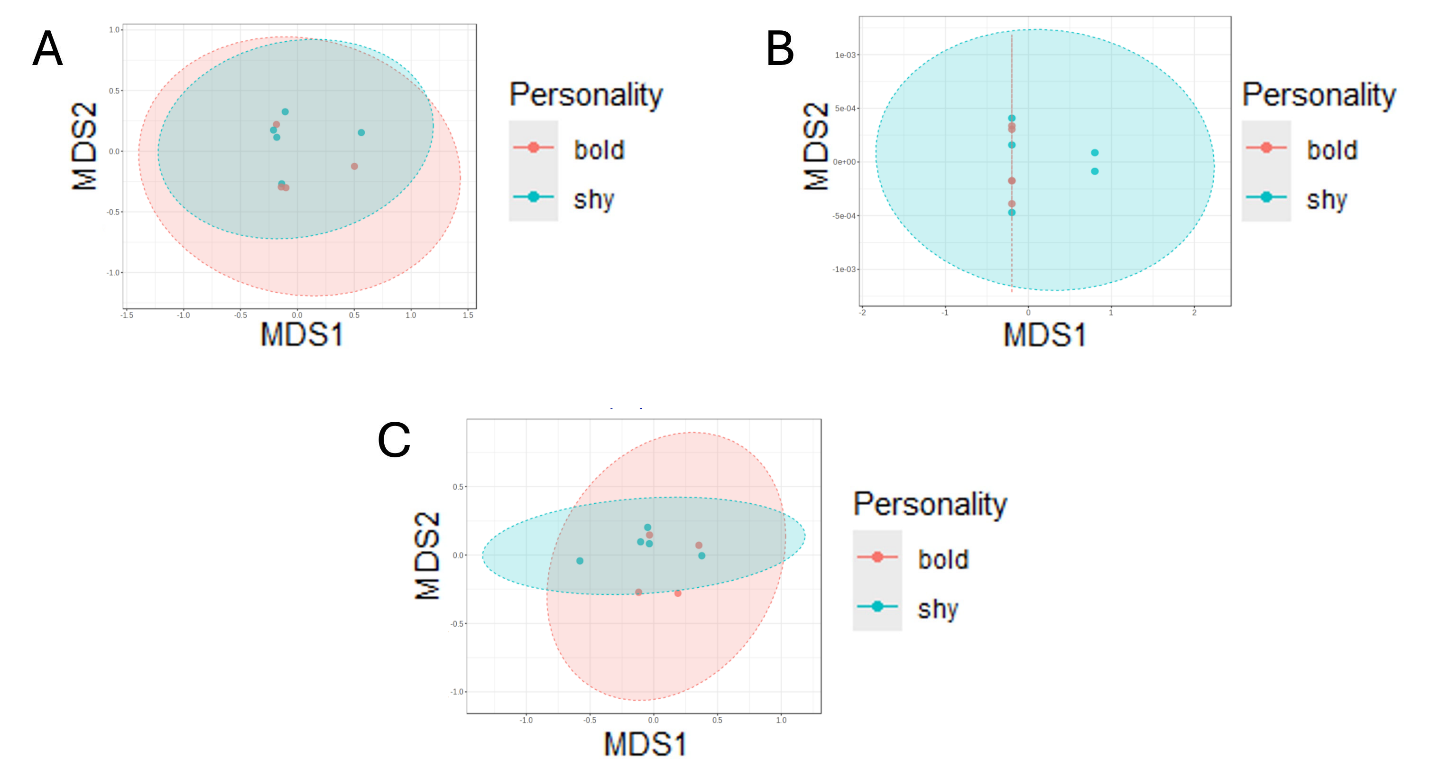


**S3 Fig.** **Non-metric multidimensional scaling of Jaccard beta diversity of bacterial communities among bold and shy individuals.** **(A)** skin samples (R^2^ = 0.11447, p = 0.595**), (B)** Oral samples (R^2^ = 0.094497 , p = 0.734), and **(C)** cloacal samples (R^2^ = 0.1137, p = 0.894). Shaded ellipses represent the 95% confidence interval.
