# Supplementary material for: The relationships between box turtle gut microbiomes and personality
Source: PLoS One. 2025 Dec 19;20(12):e0339132. doi: 10.1371/journal.pone.0339132 (PMC12716703; doi:10.1371/journal.pone.0339132)
Supplement: S4 Fig — This includes skin samples (p = 0.595), oral samples (p = 0.734), and cloacal samples (p = 0.8). (DOCX) [file pone.0339132.s004.docx]

**
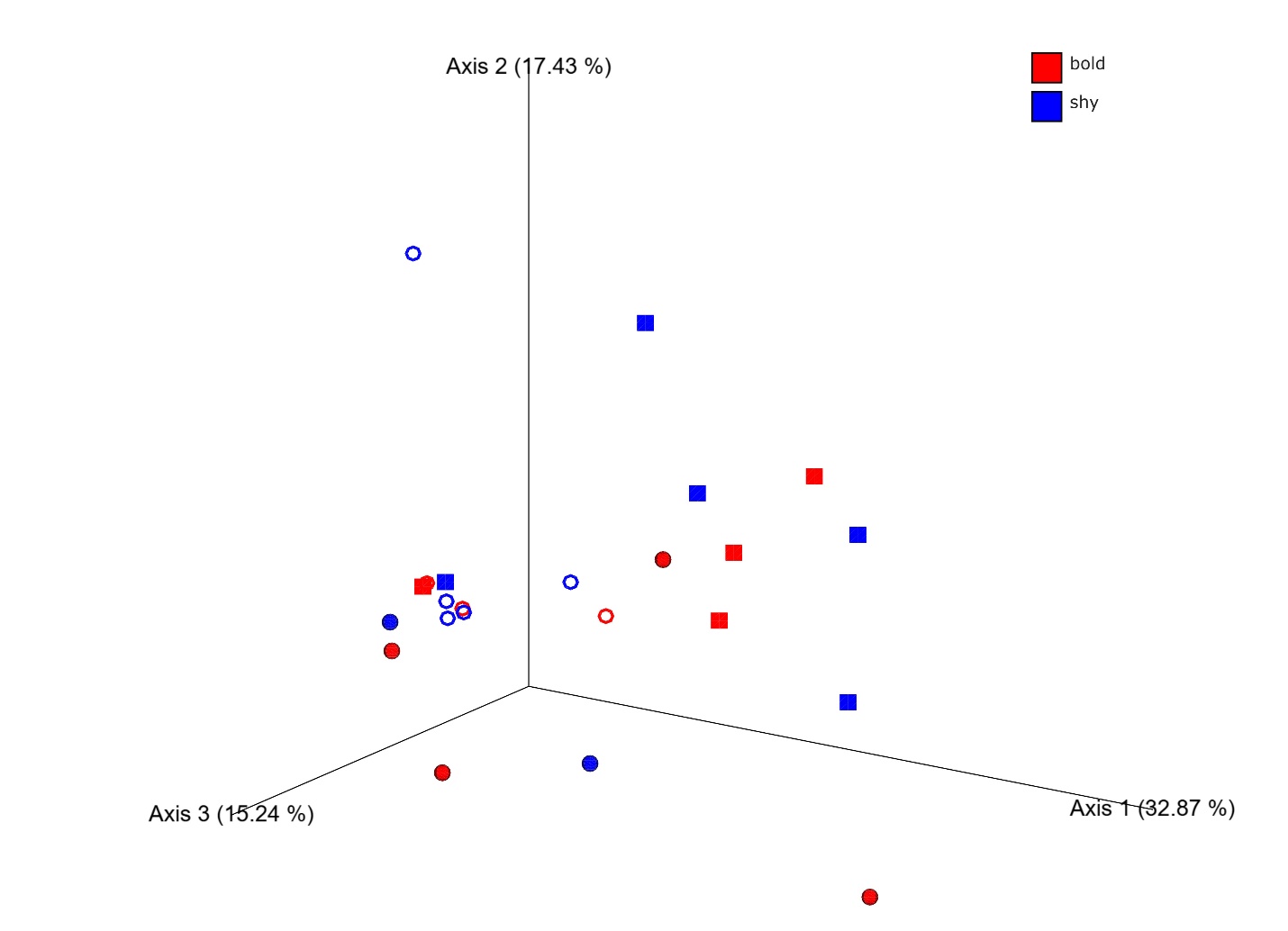

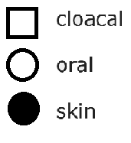
S4 Fig. Principal Coordinates Analysis of Weighted Unifrac beta diversity of bacterial communities among bold (N=6) and shy (N=5) individuals.** This includes skin samples (p = 0.595**),** oral samples (p = 0.734), and cloacal samples (p = 0.8).
