# Supplementary material for: The relationships between box turtle gut microbiomes and personality
Source: PLoS One. 2025 Dec 19;20(12):e0339132. doi: 10.1371/journal.pone.0339132 (PMC12716703; doi:10.1371/journal.pone.0339132)
Supplement: S5 Fig — This includes skin samples (p = 0.595), Oral samples (p = 0.734), and cloacal samples (p = 0.894). (DOCX) [file pone.0339132.s005.docx]

**
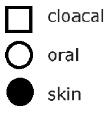
**
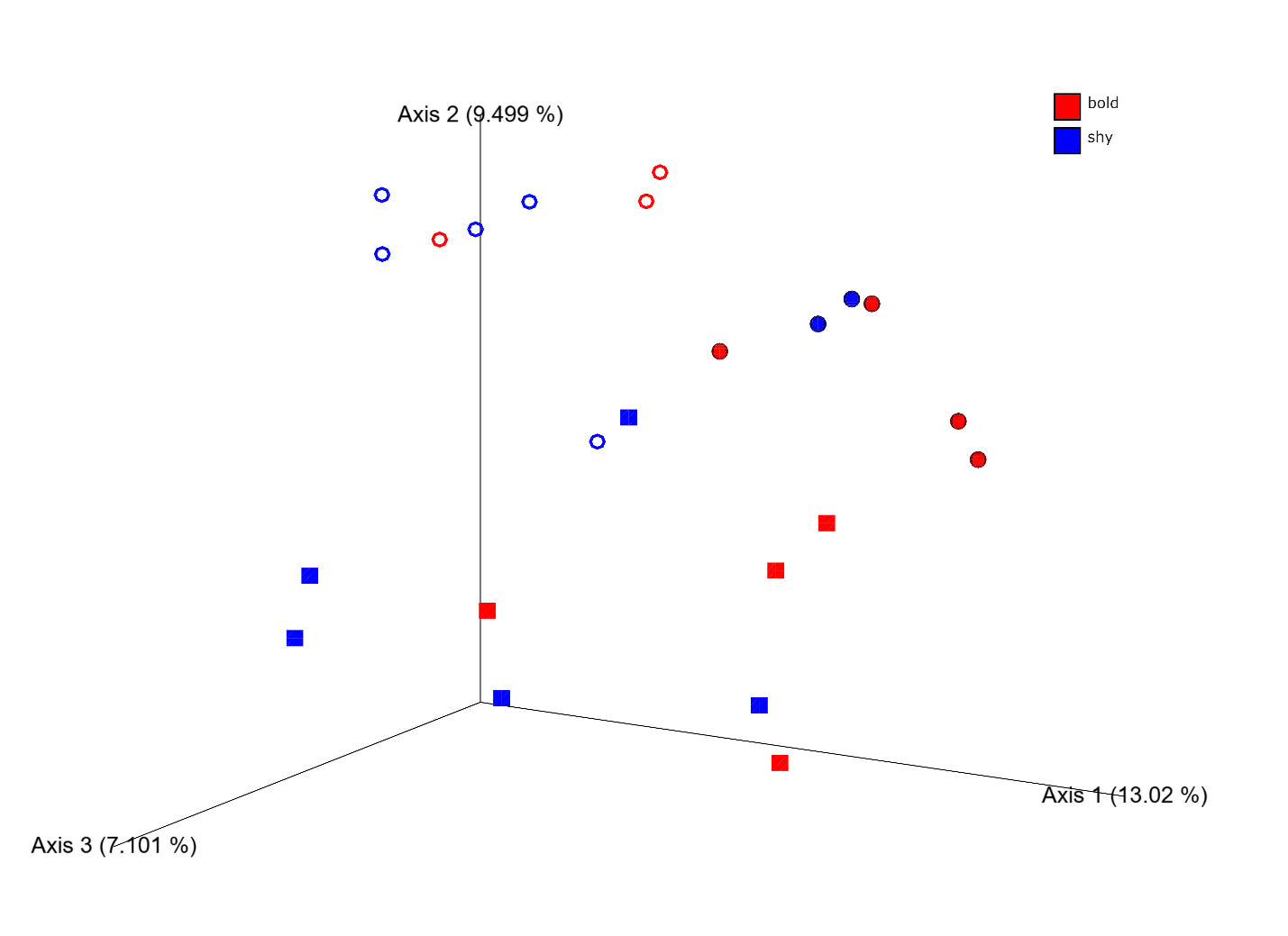


**S5 Fig. Principal Coordinates Analysis of Unweighted Unifrac beta diversity of bacterial communities among bold (N=6) and shy (N=5) individuals.** This includes skin samples (p = 0.595**),** Oral samples ( p = 0.734), and cloacal samples (p = 0.894).
