# Supplementary material for: The relationships between box turtle gut microbiomes and personality
Source: PLoS One. 2025 Dec 19;20(12):e0339132. doi: 10.1371/journal.pone.0339132 (PMC12716703; doi:10.1371/journal.pone.0339132)
Supplement: S1 Table — Faith pd: Bold_or_Shy:Sex, sum_sq (4632.50749), df (2.0), F (16.572713), PR(>F) (0.004743); Evenness: Bold_or_Shy:Sex, sum_sq (0.019633), df (2.0), F (37.233848), PR(>F) (0.00049); Shannon: Bold_or_Shy:Sex, sum_sq (14.199353), df (2.0), F (23.234192), PR(>F) (0.001921); Observed features: Bold_or_Shy:Sex, sum_sq (688441.626667), df (2.0), F (8.660089), PR(>F)(0.021627). Pairwise t-tests were performed for Faith pd (A), Evenness (B), Shannon (C), and Observed features. (DOCX) [file pone.0339132.s006.docx]

**S1 Table**: **ANOVA results on Bold/Shy and Sex (male, female, and na).**

A)

| **Comparison** | **coef** | | **std err** | | **t** | | **P>\|t\|** | | **Conf. Int. Low** | | **Conf. Int. Upp.** | | **pvalue-fdr_bh** | | **reject-fdr_bh** | |  |
| --- | --- | --- | --- | --- | --- | --- | --- | --- | --- | --- | --- | --- | --- | --- | --- | --- | --- |
| male-female | 56.5871 | | 13.21756 | | 4.281206 | | 0.00365 | | 25.33254 | | 87.84166 | | 0.007599 | | TRUE | |  |
| na-female | 15.4393 | | 14.47911 | | 1.066315 | | 0.321672 | | -18.7984 | | 49.67696 | | 0.321672 | | FALSE | |  |
| na-male | -41.1478 | | 10.23828 | | -4.01902 | | 0.005066 | | -65.3575 | | -16.9381 | | 0.007599 | | TRUE | |  |
| shy-bold | 68.55005 | | 14.47911 | | 4.73441 | | 0.002122 | | 34.31239 | | 102.7877 | | 0.002122 | | TRUE | |  |
|  | |  | |  | |  | |  | |  | |  | |  | |  | |

B)

| **Comparison** | **coef** | **std err** | **t** | **P>\|t\|** | **Conf. Int. Low** | **Conf. Int. Upp.** | **pvalue-fdr_bh** | **reject-fdr_bh** |
| --- | --- | --- | --- | --- | --- | --- | --- | --- |
| male-female | 0.129514 | 0.018154 | 7.13435 | 0.000188 | 0.086587 | 0.17244 | 0.000564 | TRUE |
| na-female | 0.118214 | 0.019886 | 5.94451 | 0.000573 | 0.07119 | 0.165237 | 0.00086 | TRUE |
| na-male | -0.0113 | 0.014062 | -0.8036 | 0.448053 | -0.04455 | 0.021951 | 0.448053 | FALSE |
| shy-bold | 0.137346 | 0.019886 | 6.906625 | 0.00023 | 0.090323 | 0.18437 | 0.00023 | TRUE |

C)

| **Comparison** | **coef** | **std err** | **t** | **P>\|t\|** | **Conf. Int. Low** | **Conf. Int. Upp.** | **pvalue-fdr_bh** | **reject-fdr_bh** |
| --- | --- | --- | --- | --- | --- | --- | --- | --- |
| shy-bold | 3.766383 | 0.677019 | 5.563183 | 0.000848 | 2.165486 | 5.367279 | 0.000848 | TRUE |
| male-female | 3.411924 | 0.618031 | 5.520633 | 0.000887 | 1.950512 | 4.873336 | 0.002661 | TRUE |
| na-female | 1.764926 | 0.677019 | 2.606906 | 0.035071 | 0.16403 | 3.365823 | 0.035071 | TRUE |
| na-male | -1.647 | 0.478725 | -3.44038 | 0.010832 | -2.779 | -0.51499 | 0.016247 | TRUE |

D)

| **Comparison** | **coef** | **std err** | **t** | **P>\|t\|** | **Conf. Int. Low** | **Conf. Int. Upp.** | **pvalue-fdr_bh** | **reject-fdr_bh** |
| --- | --- | --- | --- | --- | --- | --- | --- | --- |
| shy-bold | 857.5 | 244.176 | 3.511811 | 0.009835 | 280.1155 | 1434.885 | 0.009835 | TRUE |
| male-female | 671 | 222.9012 | 3.010302 | 0.019654 | 143.9224 | 1198.078 | 0.029785 | TRUE |
| na-female | 152.5 | 244.176 | 0.624549 | 0.552071 | -424.885 | 729.8845 | 0.552071 | FALSE |
| na-male | -518.5 | 172.6585 | -3.00031 | 0.019857 | -926.773 | -110.227 | 0.029785 | TRUE |

Faith pd: Bold_or_Shy:Sex, sum_sq (2186.8), df (1.0), F (13.191), PR(>F) (0.0109); Evenness: Bold_or_Shy:Sex, sum_sq (0.011059), df (1.0), F (35.654), PR(>F) (0.000989); Shannon: Bold_or_Shy:Sex, sum_sq (7.236), df (1.0), F (20.538), PR(>F) (0.00397); Observed features: Bold_or_Shy:Sex , sum_sq (330,851), df (1.0), F (7.115), PR(>F)(0.0372. Pairwise t-tests were performed for Faith pd (A), Evenness (B), Shannon (C), and Observed features.
