# Supplementary material for: The relationships between box turtle gut microbiomes and personality
Source: PLoS One. 2025 Dec 19;20(12):e0339132. doi: 10.1371/journal.pone.0339132 (PMC12716703; doi:10.1371/journal.pone.0339132)
Supplement: S2 Table — (DOCX) [file pone.0339132.s007.docx]

**S2 Table**. **Relative abundances of microbial phyla by sample-type and personality.**

| Sample-type | Personality | Phylum | value |
| --- | --- | --- | --- |
| Cloacal | Bold | Actinobacteria | 0.714840912 |
| Cloacal | Bold | Bacteroidetes | 0.00117252 |
| Cloacal | Bold | Chloroflexi | 0.003814819 |
| Cloacal | Bold | Firmicutes | 0.130722228 |
| Cloacal | Bold | Fusobacteria | 0.010277441 |
| Cloacal | Bold | Proteobacteria | 0.084801277 |
| Cloacal | Bold | Thermi | 0.052047782 |
| Cloacal | Bold | TM7 | 0.002323021 |
| Oral | Bold | Actinobacteria | 0.751631822 |
| Oral | Bold | Bacteroidetes | 0.148357317 |
| Oral | Bold | Chloroflexi | 0 |
| Oral | Bold | Firmicutes | 0.049041542 |
| Oral | Bold | Fusobacteria | 0 |
| Oral | Bold | Proteobacteria | 0.048949226 |
| Oral | Bold | Thermi | 0.001569373 |
| Oral | Bold | TM7 | 0.00045072 |
| Skin | Bold | Actinobacteria | 0.790182006 |
| Skin | Bold | Bacteroidetes | 0.004172775 |
| Skin | Bold | Chloroflexi | 0.001048636 |
| Skin | Bold | Firmicutes | 0.000504361 |
| Skin | Bold | Fusobacteria | 0 |
| Skin | Bold | Proteobacteria | 0.05318292 |
| Skin | Bold | Thermi | 0.138496205 |
| Skin | Bold | TM7 | 0.012413097 |
| Cloacal | Shy | Actinobacteria | 0.683900788 |
| Cloacal | Shy | Bacteroidetes | 0.042564054 |
| Cloacal | Shy | Chloroflexi | 0.002972109 |
| Cloacal | Shy | Firmicutes | 0.082706025 |
| Cloacal | Shy | Fusobacteria | 0.00645478 |
| Cloacal | Shy | Proteobacteria | 0.080639114 |
| Cloacal | Shy | Thermi | 0.098084531 |
| Cloacal | Shy | TM7 | 0.002678598 |
| Oral | Shy | Actinobacteria | 0.84215139 |
| Oral | Shy | Bacteroidetes | 0.061720391 |
| Oral | Shy | Chloroflexi | 0 |
| Oral | Shy | Firmicutes | 0.052308062 |
| Oral | Shy | Fusobacteria | 0 |
| Oral | Shy | Proteobacteria | 0.036088601 |
| Oral | Shy | Thermi | 0.007179302 |
| Oral | Shy | TM7 | 0.000552254 |
| Skin | Shy | Actinobacteria | 0.800305844 |
| Skin | Shy | Bacteroidetes | 0.018421002 |
| Skin | Shy | Chloroflexi | 0.001478031 |
| Skin | Shy | Firmicutes | 0.002536967 |
| Skin | Shy | Fusobacteria | 0 |
| Skin | Shy | Proteobacteria | 0.025014556 |
| Skin | Shy | Thermi | 0.151904485 |
| Skin | Shy | TM7 | 0.000339115 |
