# Supplementary material for: The relationships between box turtle gut microbiomes and personality
Source: PLoS One. 2025 Dec 19;20(12):e0339132. doi: 10.1371/journal.pone.0339132 (PMC12716703; doi:10.1371/journal.pone.0339132)
Supplement: S3 Table — (DOCX) [file pone.0339132.s008.docx]

**S3 Table**: **Relative abundances of microbial phyla by individual turtle.**

| IndID | Sample-type | Personality | Phylum | value |
| --- | --- | --- | --- | --- |
| TCC_AMW_cloacal_1082 | cloacal | bold | Actinobacteria | 0.7313062 |
| TCC_AMW_cloacal_4010 | cloacal | shy | Actinobacteria | 0.6286585 |
| TCC_AMW_cloacal_9872 | cloacal | bold | Actinobacteria | 0.6190891 |
| TCC_AMW_cloacal_9961 | cloacal | bold | Actinobacteria | 0.8180642 |
| TCC_AMW_cloacal_AMW_2002 | cloacal | bold | Actinobacteria | 0.7419346 |
| TCC_AMW_cloacal_AMW_51 | cloacal | shy | Actinobacteria | 0.6327976 |
| TCC_AMW_cloacal_SC_113 | cloacal | shy | Actinobacteria | 0.7833347 |
| TCC_AMW_cloacal_SC_179 | cloacal | shy | Actinobacteria | 0.7805272 |
| TCC_AMW_cloacal_SC_187 | cloacal | shy | Actinobacteria | 0.85157 |
| TCC_AMW_oral_1082 | oral | bold | Actinobacteria | 0.8610229 |
| TCC_AMW_oral_4010 | oral | shy | Actinobacteria | 0.9492386 |
| TCC_AMW_oral_9872 | oral | bold | Actinobacteria | 0.3172356 |
| TCC_AMW_oral_9961 | oral | bold | Actinobacteria | 0.9283642 |
| TCC_AMW_oral_AMW_1044 | oral | bold | Actinobacteria | 0.7362955 |
| TCC_AMW_oral_AMW_2002 | oral | bold | Actinobacteria | 0.6487628 |
| TCC_AMW_oral_AMW_51 | oral | shy | Actinobacteria | 0.7376609 |
| TCC_AMW_oral_SC_113 | oral | shy | Actinobacteria | 0.8288029 |
| TCC_AMW_oral_SC_179 | oral | shy | Actinobacteria | 0.9481982 |
| TCC_AMW_oral_SC_187 | oral | shy | Actinobacteria | 0 |
| TCC_AMW_skin_1082 | skin | bold | Actinobacteria | 0.8227094 |
| TCC_AMW_skin_4010 | skin | shy | Actinobacteria | 0.8225983 |
| TCC_AMW_skin_9872 | skin | bold | Actinobacteria | 0.8716019 |
| TCC_AMW_skin_9961 | skin | bold | Actinobacteria | 0.6713547 |
| TCC_AMW_skin_AMW_51 | skin | shy | Actinobacteria | 0.7729097 |
| TCC_AMW_skin_SC_113 | skin | shy | Actinobacteria | 0.8910773 |
| TCC_AMW_skin_SC_179 | skin | shy | Actinobacteria | 0.7323959 |
| TCC_AMW_skin_SC_187 | skin | shy | Actinobacteria | 1 |
| TCC_AMW_skin_WL_30 | skin | bold | Actinobacteria | 1 |
| TCC_AMW_cloacal_1082 | cloacal | bold | Bacteroidetes | 0 |
| TCC_AMW_cloacal_4010 | cloacal | shy | Bacteroidetes | 0.145953268 |
| TCC_AMW_cloacal_9872 | cloacal | bold | Bacteroidetes | 0 |
| TCC_AMW_cloacal_9961 | cloacal | bold | Bacteroidetes | 0 |
| TCC_AMW_cloacal_AMW_2002 | cloacal | bold | Bacteroidetes | 0.005285504 |
| TCC_AMW_cloacal_AMW_51 | cloacal | shy | Bacteroidetes | 0.07452805 |
| TCC_AMW_cloacal_SC_113 | cloacal | shy | Bacteroidetes | 0 |
| TCC_AMW_cloacal_SC_179 | cloacal | shy | Bacteroidetes | 0 |
| TCC_AMW_cloacal_SC_187 | cloacal | shy | Bacteroidetes | 0 |
| TCC_AMW_oral_1082 | oral | bold | Bacteroidetes | 0.117476193 |
| TCC_AMW_oral_4010 | oral | shy | Bacteroidetes | 0.004070491 |
| TCC_AMW_oral_9872 | oral | bold | Bacteroidetes | 0.594519445 |
| TCC_AMW_oral_9961 | oral | bold | Bacteroidetes | 0.014485997 |
| TCC_AMW_oral_AMW_1044 | oral | bold | Bacteroidetes | 0 |
| TCC_AMW_oral_AMW_2002 | oral | bold | Bacteroidetes | 0 |
| TCC_AMW_oral_AMW_51 | oral | shy | Bacteroidetes | 0.148158083 |
| TCC_AMW_oral_SC_113 | oral | shy | Bacteroidetes | 0 |
| TCC_AMW_oral_SC_179 | oral | shy | Bacteroidetes | 0 |
| TCC_AMW_oral_SC_187 | oral | shy | Bacteroidetes | 0 |
| TCC_AMW_skin_1082 | skin | bold | Bacteroidetes | 0 |
| TCC_AMW_skin_4010 | skin | shy | Bacteroidetes | 0.020948569 |
| TCC_AMW_skin_9872 | skin | bold | Bacteroidetes | 0 |
| TCC_AMW_skin_9961 | skin | bold | Bacteroidetes | 0.003129285 |
| TCC_AMW_skin_AMW_51 | skin | shy | Bacteroidetes | 0.039842506 |
| TCC_AMW_skin_SC_113 | skin | shy | Bacteroidetes | 0.00083243 |
| TCC_AMW_skin_SC_179 | skin | shy | Bacteroidetes | 0.004340104 |
| TCC_AMW_skin_SC_187 | skin | shy | Bacteroidetes | 0 |
| TCC_AMW_skin_WL_30 | skin | bold | Bacteroidetes | 0 |
| TCC_AMW_cloacal_1082 | cloacal | bold | Chloroflexi | 0.00621579 |
| TCC_AMW_cloacal_4010 | cloacal | shy | Chloroflexi | 0.003483141 |
| TCC_AMW_cloacal_9872 | cloacal | bold | Chloroflexi | 0 |
| TCC_AMW_cloacal_AMW_2002 | cloacal | bold | Chloroflexi | 0.011928487 |
| TCC_AMW_cloacal_AMW_51 | cloacal | shy | Chloroflexi | 0.003103591 |
| TCC_AMW_cloacal_SC_113 | cloacal | shy | Chloroflexi | 0 |
| TCC_AMW_cloacal_SC_179 | cloacal | shy | Chloroflexi | 0.002213831 |
| TCC_AMW_cloacal_SC_187 | cloacal | shy | Chloroflexi | 0.00526629 |
| TCC_AMW_oral_1082 | oral | bold | Chloroflexi | 0 |
| TCC_AMW_oral_4010 | oral | shy | Chloroflexi | 0 |
| TCC_AMW_oral_9872 | oral | bold | Chloroflexi | 0 |
| TCC_AMW_oral_9961 | oral | bold | Chloroflexi | 0 |
| TCC_AMW_oral_AMW_1044 | oral | bold | Chloroflexi | 0 |
| TCC_AMW_oral_AMW_2002 | oral | bold | Chloroflexi | 0 |
| TCC_AMW_oral_AMW_51 | oral | shy | Chloroflexi | 0 |
| TCC_AMW_oral_SC_113 | oral | shy | Chloroflexi | 0 |
| TCC_AMW_oral_SC_179 | oral | shy | Chloroflexi | 0 |
| TCC_AMW_oral_SC_187 | oral | shy | Chloroflexi | 0 |
| TCC_AMW_skin_1082 | skin | bold | Chloroflexi | 0.004827788 |
| TCC_AMW_skin_4010 | skin | shy | Chloroflexi | 0.003360019 |
| TCC_AMW_skin_9872 | skin | bold | Chloroflexi | 0 |
| TCC_AMW_skin_9961 | skin | bold | Chloroflexi | 0 |
| TCC_AMW_skin_AMW_51 | skin | shy | Chloroflexi | 0.000239949 |
| TCC_AMW_skin_SC_113 | skin | shy | Chloroflexi | 0.002560116 |
| TCC_AMW_skin_SC_179 | skin | shy | Chloroflexi | 0 |
| TCC_AMW_skin_SC_187 | skin | shy | Chloroflexi | 0 |
| TCC_AMW_skin_WL_30 | skin | bold | Chloroflexi | 0 |
| TCC_AMW_cloacal_1082 | cloacal | bold | Firmicutes | 0.126774283 |
| TCC_AMW_cloacal_4010 | cloacal | shy | Firmicutes | 0.135648977 |
| TCC_AMW_cloacal_9872 | cloacal | bold | Firmicutes | 0.1507657 |
| TCC_AMW_cloacal_9961 | cloacal | bold | Firmicutes | 0.028218923 |
| TCC_AMW_cloacal_AMW_2002 | cloacal | bold | Firmicutes | 0.165843514 |
| TCC_AMW_cloacal_AMW_51 | cloacal | shy | Firmicutes | 0.135045455 |
| TCC_AMW_cloacal_SC_113 | cloacal | shy | Firmicutes | 0.061578562 |
| TCC_AMW_cloacal_SC_179 | cloacal | shy | Firmicutes | 0.000714139 |
| TCC_AMW_cloacal_SC_187 | cloacal | shy | Firmicutes | 0.0737461 |
| TCC_AMW_oral_1082 | oral | bold | Firmicutes | 0.002754986 |
| TCC_AMW_oral_4010 | oral | shy | Firmicutes | 0.030169524 |
| TCC_AMW_oral_9872 | oral | bold | Firmicutes | 0.060110489 |
| TCC_AMW_oral_9961 | oral | bold | Firmicutes | 0.017041334 |
| TCC_AMW_oral_AMW_1044 | oral | bold | Firmicutes | 0.215453402 |
| TCC_AMW_oral_AMW_2002 | oral | bold | Firmicutes | 0.10261194 |
| TCC_AMW_oral_AMW_51 | oral | shy | Firmicutes | 0.061695279 |
| TCC_AMW_oral_SC_113 | oral | shy | Firmicutes | 0.068478829 |
| TCC_AMW_oral_SC_179 | oral | shy | Firmicutes | 0 |
| TCC_AMW_oral_SC_187 | oral | shy | Firmicutes | 0.576054217 |
| TCC_AMW_skin_1082 | skin | bold | Firmicutes | 0.00129577 |
| TCC_AMW_skin_4010 | skin | shy | Firmicutes | 0.002668608 |
| TCC_AMW_skin_9872 | skin | bold | Firmicutes | 0.000376292 |
| TCC_AMW_skin_9961 | skin | bold | Firmicutes | 0.000632889 |
| TCC_AMW_skin_AMW_51 | skin | shy | Firmicutes | 0 |
| TCC_AMW_skin_SC_113 | skin | shy | Firmicutes | 0.00899967 |
| TCC_AMW_skin_SC_179 | skin | shy | Firmicutes | 0 |
| TCC_AMW_skin_SC_187 | skin | shy | Firmicutes | 0 |
| TCC_AMW_skin_WL_30 | skin | bold | Firmicutes | 0 |
| TCC_AMW_cloacal_1082 | cloacal | bold | Proteobacteria | 0.068837555 |
| TCC_AMW_cloacal_4010 | cloacal | shy | Proteobacteria | 0.084611291 |
| TCC_AMW_cloacal_9872 | cloacal | bold | Proteobacteria | 0.045902418 |
| TCC_AMW_cloacal_9961 | cloacal | bold | Proteobacteria | 0.120273136 |
| TCC_AMW_cloacal_AMW_2002 | cloacal | bold | Proteobacteria | 0.074459175 |
| TCC_AMW_cloacal_AMW_51 | cloacal | shy | Proteobacteria | 0.073716825 |
| TCC_AMW_cloacal_SC_113 | cloacal | shy | Proteobacteria | 0.028182781 |
| TCC_AMW_cloacal_SC_179 | cloacal | shy | Proteobacteria | 0.02244182 |
| TCC_AMW_cloacal_SC_187 | cloacal | shy | Proteobacteria | 0.031958447 |
| TCC_AMW_oral_1082 | oral | bold | Proteobacteria | 0.017757681 |
| TCC_AMW_oral_4010 | oral | shy | Proteobacteria | 0.008931137 |
| TCC_AMW_oral_9872 | oral | bold | Proteobacteria | 0.022353931 |
| TCC_AMW_oral_9961 | oral | bold | Proteobacteria | 0.038226458 |
| TCC_AMW_oral_AMW_1044 | oral | bold | Proteobacteria | 0.044897769 |
| TCC_AMW_oral_AMW_2002 | oral | bold | Proteobacteria | 0.248625295 |
| TCC_AMW_oral_AMW_51 | oral | shy | Proteobacteria | 0.042739628 |
| TCC_AMW_oral_SC_113 | oral | shy | Proteobacteria | 0.102718244 |
| TCC_AMW_oral_SC_179 | oral | shy | Proteobacteria | 0.051801802 |
| TCC_AMW_oral_SC_187 | oral | shy | Proteobacteria | 0.423945783 |
| TCC_AMW_skin_1082 | skin | bold | Proteobacteria | 0.077328206 |
| TCC_AMW_skin_4010 | skin | shy | Proteobacteria | 0.03209607 |
| TCC_AMW_skin_9872 | skin | bold | Proteobacteria | 0.05250647 |
| TCC_AMW_skin_9961 | skin | bold | Proteobacteria | 0.052951725 |
| TCC_AMW_skin_AMW_51 | skin | shy | Proteobacteria | 0.020613834 |
| TCC_AMW_skin_SC_113 | skin | shy | Proteobacteria | 0.014763857 |
| TCC_AMW_skin_SC_179 | skin | shy | Proteobacteria | 0.031288811 |
| TCC_AMW_skin_SC_187 | skin | shy | Proteobacteria | 0 |
| TCC_AMW_skin_WL_30 | skin | bold | Proteobacteria | 0 |
| TCC_AMW_cloacal_1082 | cloacal | bold | Thermi | 0.062784117 |
| TCC_AMW_cloacal_4010 | cloacal | shy | Thermi | 0.001644816 |
| TCC_AMW_cloacal_9872 | cloacal | bold | Thermi | 0.184242808 |
| TCC_AMW_cloacal_9961 | cloacal | bold | Thermi | 0.028167193 |
| TCC_AMW_cloacal_AMW_2002 | cloacal | bold | Thermi | 0.000548768 |
| TCC_AMW_cloacal_AMW_51 | cloacal | shy | Thermi | 0.076762845 |
| TCC_AMW_cloacal_SC_113 | cloacal | shy | Thermi | 0.126415248 |
| TCC_AMW_cloacal_SC_179 | cloacal | shy | Thermi | 0.194102997 |
| TCC_AMW_cloacal_SC_187 | cloacal | shy | Thermi | 0.031922376 |
| TCC_AMW_oral_1082 | oral | bold | Thermi | 0.000658801 |
| TCC_AMW_oral_4010 | oral | shy | Thermi | 0.006488842 |
| TCC_AMW_oral_9872 | oral | bold | Thermi | 0.005780558 |
| TCC_AMW_oral_9961 | oral | bold | Thermi | 0.001881971 |
| TCC_AMW_oral_AMW_1044 | oral | bold | Thermi | 0 |
| TCC_AMW_oral_AMW_2002 | oral | bold | Thermi | 0 |
| TCC_AMW_oral_AMW_51 | oral | shy | Thermi | 0.009746066 |
| TCC_AMW_oral_SC_113 | oral | shy | Thermi | 0 |
| TCC_AMW_oral_SC_179 | oral | shy | Thermi | 0 |
| TCC_AMW_oral_SC_187 | oral | shy | Thermi | 0 |
| TCC_AMW_skin_1082 | skin | bold | Thermi | 0.078519478 |
| TCC_AMW_skin_4010 | skin | shy | Thermi | 0.118328481 |
| TCC_AMW_skin_9872 | skin | bold | Thermi | 0.075515336 |
| TCC_AMW_skin_9961 | skin | bold | Thermi | 0.224675644 |
| TCC_AMW_skin_AMW_51 | skin | shy | Thermi | 0.166393997 |
| TCC_AMW_skin_SC_113 | skin | shy | Thermi | 0.081766637 |
| TCC_AMW_skin_SC_179 | skin | shy | Thermi | 0.230559673 |
| TCC_AMW_skin_SC_187 | skin | shy | Thermi | 0 |
| TCC_AMW_skin_WL_30 | skin | bold | Thermi | 0 |
| TCC_AMW_cloacal_1082 | cloacal | bold | TM7 | 0.004082011 |
| TCC_AMW_cloacal_4010 | cloacal | shy | TM7 | 0 |
| TCC_AMW_cloacal_9872 | cloacal | bold | TM7 | 0 |
| TCC_AMW_cloacal_9961 | cloacal | bold | TM7 | 0.005276499 |
| TCC_AMW_cloacal_AMW_2002 | cloacal | bold | TM7 | 0 |
| TCC_AMW_cloacal_AMW_51 | cloacal | shy | TM7 | 0.004045659 |
| TCC_AMW_cloacal_SC_113 | cloacal | shy | TM7 | 0.000488719 |
| TCC_AMW_cloacal_SC_179 | cloacal | shy | TM7 | 0 |
| TCC_AMW_cloacal_SC_187 | cloacal | shy | TM7 | 0.005536819 |
| TCC_AMW_oral_1082 | oral | bold | TM7 | 0.000329401 |
| TCC_AMW_oral_4010 | oral | shy | TM7 | 0.001101427 |
| TCC_AMW_oral_9872 | oral | bold | TM7 | 0 |
| TCC_AMW_oral_9961 | oral | bold | TM7 | 0 |
| TCC_AMW_oral_AMW_1044 | oral | bold | TM7 | 0.00335336 |
| TCC_AMW_oral_AMW_2002 | oral | bold | TM7 | 0 |
| TCC_AMW_oral_AMW_51 | oral | shy | TM7 | 0 |
| TCC_AMW_oral_SC_113 | oral | shy | TM7 | 0 |
| TCC_AMW_oral_SC_179 | oral | shy | TM7 | 0 |
| TCC_AMW_oral_SC_187 | oral | shy | TM7 | 0 |
| TCC_AMW_skin_1082 | skin | bold | TM7 | 0.015319345 |
| TCC_AMW_skin_4010 | skin | shy | TM7 | 0 |
| TCC_AMW_skin_9872 | skin | bold | TM7 | 0 |
| TCC_AMW_skin_9961 | skin | bold | TM7 | 0.047255722 |
| TCC_AMW_skin_AMW_51 | skin | shy | TM7 | 0 |
| TCC_AMW_skin_SC_113 | skin | shy | TM7 | 0 |
| TCC_AMW_skin_SC_179 | skin | shy | TM7 | 0.001415542 |
| TCC_AMW_skin_SC_187 | skin | shy | TM7 | 0 |
| TCC_AMW_skin_WL_30 | skin | bold | TM7 | 0 |
